# Characterization and description of *Gabonibacter chumensis* sp. nov., isolated from feces of a patient with non-small cell lung cancer treated with immunotherapy

**DOI:** 10.1007/s00203-023-03671-0

**Published:** 2023-09-24

**Authors:** Khoudia Diop, Reilly Pidgeon, Awa Diop, Myriam Benlaïfaoui, Wiam Belkaid, Julie Malo, Eve Bernet, Frederic Veyrier, Maxime Jacq, Yves Brun, Arielle Elkrief, Bastien Castagner, Bertrand Routy, Corentin Richard

**Affiliations:** 1 Laboratory of Immunotherapy and Onco-Microbiome, University of Montreal Healthcare Research Center (CRCHUM), 900 Rue Saint-Denis, Montreal, QC H2X 0A9 Canada; 2 Department of Pharmacology & Therapeutics, Faculty of Medicine and Health Sciences, McGill University, 3655 Promenade Sir-William-Osler, Montreal, QC H3G 1Y6 Canada; 3 Department of Biology, University of North Carolina Greensboro, 321 McIver Street, PO Box 26170, Greensboro, NC 27402 USA; 4 INRS-Centre Armand-Frappier Santé Biotechnologie, Bacterial Symbionts Evolution, Laval, QC H7V 1B7 Canada; 5 Faculty of Medicine, Department of Microbiology and Immunology, University of Montreal, Montreal, QC Canada; 6 Hematology-Oncology Service, Department of Medicine, University of Montreal Healthcare Centre (CHUM), Montreal, QC H2X 0A9 Canada

**Keywords:** Bacteria, Cancer, *Gabonibacter chumensis*, Gut microbiota, Immunotherapy, Non-small cell lung cancer

## Abstract

A polyphasic taxonomic approach, incorporating analysis of phenotypic features, cellular fatty acid profiles, 16S rRNA gene sequences, and determination of average nucleotide identity (ANI) plus digital DNA–DNA hybridization (dDDH), was applied to characterize an anaerobic bacterial strain designated KD22^T^ isolated from human feces. 16S rRNA gene-based phylogenetic analysis showed that strain KD22^T^ was found to be most closely related to species of the genus *Gabonibacter.* At the 16S rRNA gene level, the closest species from the strain KD22^T^ corresponded with *Gabonibacter massiliensis* GM7^T^, with a similarity of 97.58%. Cells of strain KD22^T^ were Gram-negative coccobacillus, positive for indole and negative for catalase, nitrate reduction, oxidase, and urease activities. The fatty acid analysis demonstrated the presence of a high concentration of iso-C_15: 0_ (51.65%). Next, the complete whole-genome sequence of strain KD22^T^ was 3,368,578 bp long with 42 mol% of DNA G + C contents. The DDH and ANI values between KD22^T^ and type strains of phylogenetically related species were 67.40% and 95.43%, respectively. These phylogenetic, phenotypic, and genomic results supported the affiliation of strain KD22^T^ as a novel bacterial species within the genus *Gabonibacter.* The proposed name is* Gabonibacter chumensis* and the type strain is KD22^T^ (= CSUR Q8104^T^ = DSM 115208^ T^).

**Supplementary Information:**

The online version contains supplementary material available at 10.1007/s00203-023-03671-0.

## Introduction

Despite recent therapeutic advances, lung cancer remains the number one cause of cancer-related death worldwide (He et al. 2021). According to statistics Canada, more Canadians died from lung cancer in 2021 (approximately 21,000 Canadians) than from breast, colorectal, and pancreatic cancers combined. Within lung cancer, non-small cell lung cancer (NSCLC) represents the most common histology with about 85% of all lung cancer (Remark et al. 2015) and they are most often diagnosed in the advanced stage (He et al. 2021). Over the past decade, immunotherapy has demonstrated superior efficacy compared to chemotherapy and is now incorporated in the standard-of-care in NSCLC (Rocha and Arriola 2019). Recently, the gut microbiota has emerged as one of the key regulators of immune checkpoint inhibitors efficacy and modulation of the microbiota are currently being evaluated in the immuno-oncology arena (Gopalakrishnan et al. 2018; Li et al. 2019; Derosa et al. 2021). The study of the gut microbiota is limited by challenges with current sequencing methodologies, so we applied culturomics technique to isolate different species.

*Gabonibacter massiliensis* is currently the only validly published species of the genus *Gabonibacter* (https://lpsn.dsmz.de/genus/gabonibacter) and wa*s* first cultured from a human fecal sample of a healthy young Gabonese (Mourembou et al. 2016). It belongs to the family of *Porphyromonadaceae* within the *Bacteroidota* phylum (Krieg et al. 2010). The Genus *Gabonibacter* regroups Gram-negative anaerobic coccobacillus bacterium that exhibited neither catalase nor oxidase activities (Mourembou et al. 2016). During a study addressing the gut microbiota composition of NSCLC patients treated with anti-PD-1 immunotherapy, using the culturomics approach (Dubourg et al. 2014; Lagier et al. 2015, 2016), we recovered a previously unknown bacterial species designated strain KD22^T^. In this paper, using a polyphasic taxonomic approach combining analysis of phylogenic identification, phenotypic and biochemical specificities, and genomic features, we determined the taxonomic affiliation of strain KD22^T^ isolated from a fecal sample of an NSCLC patient.

## Materials and methods

### Bacterial strain isolation and identification

Strain KD22^T^ was isolated from a fecal sample collected from a patient with advanced NSCLC treated with anti-PD-1 immunotherapy, at the University of Montreal Healthcare Centre (CHUM; Montreal/Canada). The stool samples collected were stored at −80 ºC in November 2019 prior to initiating culturomics. The patient had not received any antibiotics treatment within the last 3 months before fecal collection. He gave a signed informed consent at the time of sampling and the study was approved by CHUM Research Ethics Committee 20.300. To isolate gut bacteria, 1 g of the fecal sample was injected into an anaerobic culture bottle (BACTEC Lytic/10 Anaerobic/F Culture Vials) enriched with 4 ml filter-sterilized rumen fluid and 5% sheep blood (Cedarlane Labs, Burlington, Canada), and then incubated at 37 ºC. After 7 days of incubation, 100 µl culture broth was sampled and plated on sheep blood-enriched Columbia agar (BioMérieux). The agar plates were incubated in an anaerobic chamber (5% H_2_, 5% CO_2_, and 90% N_2_) at 37 ºC for 48 h. Each emerging colony was purified and identified using MALDI-TOF mass spectrometry (MS) with a Microflex LT spectrometer (Bruker, Daltonics, Germany) that compared the spectra with those present in the library (Bruker database and CRCHUM database, constantly updated), as previously reported as previously described (Seng et al. 2009, 2013). When the score was < 1.7, no identification was considered reliable.

The MALDI-TOF MS identification of strain KD22^T^ was not successful. Therefore, to achieve the identification and determination of phylogenetic affiliation of strain KD22^T^, its 16S rRNA gene was sequenced as previously described using fd1, and rp2 primers and a 3730xl DNA Analyzer from Applied Biosystems™ (Technelysium Pty. Ltd) (Routy et al. 2022). Obtained 16S rRNA gene sequence was assembled and corrected using ChromasPro software (http://technelysium.com.au/wp/chromaspro/). Phylogenetic neighbors of KD22^T^ were identified using the BLASTn program (Altschul et al. 1997) and the nucleotide collection (nr/nt) of the NCBI database (Yoon et al. 2017), available at https://blast.ncbi.nlm.nih.gov/Blast.cgi. Based on the BLAST results, the 16S rRNA gene sequences of closest relatives validly published were extracted from the GenBank database and aligned using the CLUSTAL W tool (Thompson et al. 1994; Higgins et al. 1996) integrated into the MEGAX program (Kumar et al. 2018). Phylogenetic interferences were reconstructed using the neighbor-joining method (Saitou and Nei 1987) with the maximum composite likelihood model and bootstrap values of 100 replicates using MEGAX software (available at https://www.megasoftware.net/).

### Morphologic and phenotypic characteristics

Strain KD22^T^ cell morphology was assessed by transmission electron microscopic as previously reported (Routy et al. 2022). Gram stain was assessed using the standard protocol. The bacterium motility was investigated using a Leica DM 1000 photonic microscope (Leica Microsystems) at 100 X magnification. To test sporulation, a thermal shock at 80 °C for 20 min of strain KD22^T^ was performed. The growth temperature range was determined by culturing strain KD22^T^ on Columbia agar and incubated for 2 days at various temperatures (room, 28, 37, 42, and 56 °C) under different atmospheres (anaerobic, microaerophilic, and aerobic conditions). The pH range growth was also tested at pH 5, 6, 6.5, 7, 7.5, and 8.5. Tolerance of NaCl was determined for concentrations ranked between 0 and 100 g/l. Catalase and oxidase productions were also detected (BioMérieux). Enzymatic and biochemical properties of strain KD22^T^ were determined in duplicate using the API^®^ 20A, API^®^ ZYM, and Rapid ID 32A identification systems (BioMérieux). Short-chain fatty acids were analyzed using both a gas chromatograph (Hewlett Packard) and Microbial Identification System software.

### Genome sequencing and annotation

Genomic DNA was sequenced using MiSeq Illumina. Libraries were generated using the NxSeq^®^ AmpFREE Low DNA Library Kit Library Preparation Kit (Lucigen) according to the manufacturer’s recommendations, with 700 ng of genomic DNA as starting material. Dual-indexed adaptors were purchased from IDT. Libraries were quantified using the Kapa Illumina GA with Revised Primers-SYBR Fast Universal kit (Kapa Biosystems). The average size fragment was determined using a LabChip GX II (PerkinElmer) instrument. The libraries were normalized and pooled, denatured in 0.05 N NaOH, and neutralized using HT1 buffer. The pool was loaded at 225 pm on an Illumina NovaSeq S4 lane using Xp protocol as per the manufacturer’s recommendations. The run was performed for 2X150 cycles (paired-end mode). A phiX library was used as a control and mixed with libraries at 1% level. Base calling was performed with RTA v3. Program bcl2fastq2 v2.20 was then used to demultiplex samples and generate fastq reads. Genome assembly was performed as previously reported (Routy et al. 2022). Then, the genome of strain KD22^T^ was compared to those of type strains of phylogenetically related species.

The core genome among all genomes compared was built using CoreCruncher software as previously described (Harris et al. 2020) with Usearch Global v8.0 (Edgar, 2010) and the stringent option. This method conservatively seeks out orthologues within large sets of whole genomes with the added ability to filter out paralogues and xenologues. Orthologs were defined with > 70% protein sequence identity and > 80% sequence length conservation and all other parameters were set to default. The core genome was defined as the set of single copy orthologs found in at least 90% of the genomes and resulted to 108 genes. Protein sequences of each core gene were then aligned using Mafft v7.407 (Katoh and Standley 2013) with default parameters. Protein alignments were then reverse translated into their corresponding nucleotide sequences. Finally, the nucleotide alignments of all the core genes of each genome were concatenated into a single large alignment as previously described (Bobay and Ochman 2017). Maximum-likelihood phylogenomic trees were built from the concatenated alignment of the core genome using FastTree v2.1.11 with GTR model (Price et al. 2010). Branch supports were evaluated by generating 100 bootstraps replicates using the same parameters. The trees were visualized with FigTree v1.4.4 (http://tree.bio.ed.ac.uk/software/figtree/). The genomic similarity among all compared genomes was evaluated by calculating two parameters: digital DNA–DNA hybridization (dDDH), average nucleotide identity (ANI), and average amino acid identity (AAI) values using Genome-to-Genome Distance Calculator (GGDC) (Auch et al. 2010) and the ANI and the AAI calculators (Goris et al. 2007), respectively.

## Results

### Strain identification and phylogenetic analysis

**Fig. 1:**
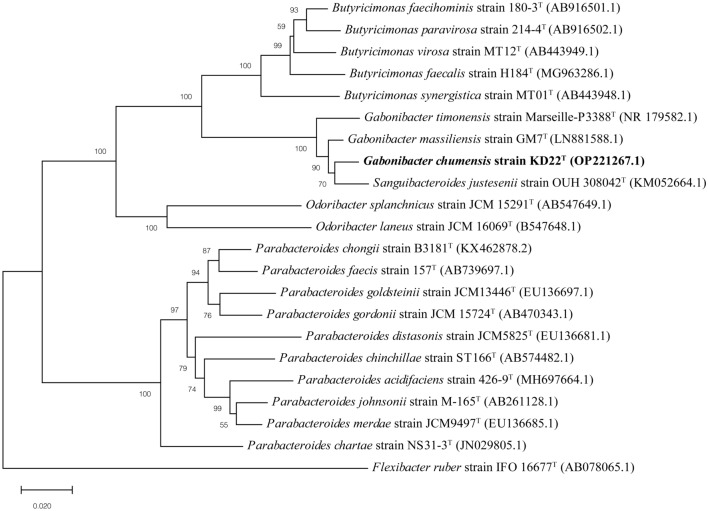
Phylogenetic tree, based on the 16S rRNA gene sequence of strain KD22^T^ and closest related taxa, constructed using the neighbor-joining method with the maximum composite likelihood model. Branch supports were evaluated by generating 100 bootstraps replicates. 16S rRNA gene GenBank accession numbers are shown in parentheses. The scale bar represented 0.02 substitutions per nucleotide position

The strain KD22^T^ could not be identified by MALDI-TOF MS, which indicated that this strain may be a new putative species (Fig. S1). Subsequently, an almost-complete 16S rRNA gene sequence of strain KD22^T^ was determined (1520 bp, GenBank accession no. OP221267.1), and database searches using nucleotide BLAST revealed highest sequence similarity to that of 2 uncultured bacteria (97.66% to *Porphyromonadaceae* bacterium AIP925^T^, and 97.46% to *Porphyromonadaceae sp.* S190), to ‘*Sanguibacteroides justesenii*’ OUH 308042^ T^ (97.72%) not validly published and to members of the genus *Gabonibacter* (97.58% to *G. massiliensis* GM7^T^ and 96.20% to ‘*Gabonibacter timonensis*’ Marseille-P3388^T^). Phylogenetic analysis of KD22^T^ and other related type strain, performed using the neighbor-joining method, confirmed that strain KD22^T^ was phylogenetically closest to *G. massiliensis* GM7^T^ detected from human feces (Fig. 1). This cluster was supported by a high bootstrap value of 100%. These results are consistent with those obtained with the maximum-likelihood (Fig. S2) methods. Based on the results of phylogenetic analysis, strain KD22^T^ was considered to be a member of the genus *Gabonibacter*.

### Phenotypical and biochemical characteristics

Cells of strain of KD22^T^ were Gram-negative, non-spore-forming coccobacilli without catalase, oxidase, and urease activities. The bacterium measured up to 0.5 µm in diameter and 1.5 µm in length (Fig. S3). Bacteria occurred as single rods or in a short chain. Bacterial colonies were circular, white to pale cream with 0.6–1.7 mm of diameter after 48 h of incubation at 37 ºC in anaerobic conditions on sheep blood-enriched Columbia agar. Growth occurred between 28 and 37 ºC with an optimal at 37 ºC. No growth was observed in the microaerophilic and aerobic atmosphere or at 45 and 55 ºC. The strain grew at a pH ranging from 6 to 7.5, with optimal growth at pH 7.0 and a NaCl concentration of less than 5%. The biochemical and enzymatic characteristics of KD22^T^, obtained using API^®^ ZYM, API^®^ 20A, and Rapid ID 32A strips, are given in the species description section and the features that differentiate strain KD22^T^ from its close neighbors are shown in Table 1. The analysis of the total cellular fatty acid composition demonstrated that saturated branch-chain iso-C_15:0_ was the major fatty acid (51.65%). The cellular fatty acid profile of strain KD22^T^ and those of its closest related species are mentioned in Table 2. However, the differences noted in the cellular fatty acid profile between studies may be due to differences in the bacterial culture conditions and the extraction method used.

**Table 1:** Differential phenotypic features between strain KD22^T^ and closest related species

Characteristic	1	2	3	4	5	6	7	8
Cell diameter (μm)	0.5 × 1.5	0.4 × 0.56	0.5 × 1.4–2.0	1.0 × 1.5	1.0 × 1.5	1.0 × 1.5	0.4–1.9 × 1.4–19.1	0.8–1.6 × 1.2–12
Motility	−	+	−	−	−	−	−	−
Nitrate reduction	−	−	−	−	−	−	−	−
Gelatin digestion	−	+	−	−	−	−	+	−
Aesculin hydrolysis	−	na	−	−	−	−	−	+
Production of								
Indole	+	+	+	+	+	+	+	−
Urease	−	−	−	−	−	−	−	−
Catalase	−	−	+	+	+	−	−	+
Enzymes activities								
Alkaline phosphatase	+	−	na	+	+	+	+	+
N-acetyl-β-glucosaminidase	−	−	+	+	+	+	+	+
β-Galactosidase	−	−	+	+	+	+	+	+
α-Glucosidase	−	na	na	−	−	−	+	+
Fermentation								
Glucose	+	+	+	+	+	+	−	+
Lactose	-	-	+	−	+	+	−	+
Mannitol	w	+	+	−	-	−	−	−
Mannose	−	−	+	−	+	−	−	+
Raffinose	+	−	na	−	−	−	−	+
Rhamnose	−	−	−	−	−	−	−	+

Strains: 1, KD22^T^; 2, *Gabonibacter massiliensis* GM7^T^; 3, *Butyricimonas faecalis* H184^T^; 4, *Butyricimonas virosa* MT12^T^, 5, *Butyricimonas faecihominis* 180-3^ T^; 6, *Butyricimonas paravirosa* 214-4^ T^; 7, *Odoribacter laneus* YIT 12061^ T^; 8, *Parabacteroides distasonis* DSM 20701^ T^*.* + , Positive; -, negative, and na, no available data. Data were obtained from the original descriptions of species

**Table 2:** Cellular fatty acid composition (%) of strain KD22^T^ compared to those of related species

Fatty acid (%)	1	2*	3*	4*	5*	6*	7*	8*
Saturated straight-chain								
C_12:0_	−	−	−	−	TR	TR	−	−
C_14:0_	2.22	1.9	1.7	1.8	TR	1.8	1.1	1.4
C_15:0_	−	−	3.3	−	TR	−	−	2.6
C_16:0_	7.42	4.3	1.8	1.9	2.8	3.2	3.4	3.2
C_17:0_	TR	−	−	−	−	−	−	−
C_18:0_	1.71	−	−	−	TR	TR	−	TR
Unsaturated straight-chain								
C_16:1ω7_	−	−	−	−	−	−	−	1.8
C_18:1ω9_	3.57	3.1	−	4.4	8.3	9.5	10.8	13.8
C_18:2ω6_	−	1.4	−	−	1.4	1.5	−	1.2
C_18:2ω9,12_	−	−	−	−	−	−	TR	−
Hydroxy acids								
iso-C_15:0_ 3-OH	2.88	−	1.0	1.5	TR	1.8	−	−
C_15:0_ 3-OH	TR	−	−	−	−	−	−	TR
iso-C_16:0_ 3-OH	TR	−	−	−	−	−	−	−
C_16:0_ 3-OH	14.90	2.7	6.4	4.0	1.7	6.3	−	4.7
C_17:0_ 3-OH	−	2.8	−	−	−	−	−	TR
iso-C_17:0_ 3-OH	8.45	−	12.8	6.8	5.3	10.6	−	23.2
anteiso-C_17:0_ 3-OH	−	−	−	−	−	−	−	4.1
Saturated branch-chain								
iso-C_5:0_	−	2.8	−	−	−	−	−	−
iso-C_11:0_	−	−	−	−	TR	TR	−	−
iso-C_13:0_	TR	−	−	−	1.0	1.0	−	TR
iso-C_13:0_	−	−	−	−	−	−	−	TR
iso-C_15:0_	51.65	75.6	63.5	74.1	64.6	57.6	52.5	6.4
antesio-C_15:0_	1.75	2.1	2.8	2.1	1.8	1.7	8.8	31.5
iso-C_17:0_	TR	−	1.2	−	1.0	TR	TR	−

Strains: 1, KD22^T^; 2, *Gabonibacter massiliensis* GM7^T^; 3, *Butyricimonas faecalis* H184^T^; 4, *Butyricimonas virosa* MT12^T^; 5, *Butyricimonas faecihominis* 180-3^ T^; 6, *Butyricimonas paravirosa* 214-4^ T^; 7, *Odoribacter laneus* YIT 12061^ T^; 8, *Parabacteroides distasonis* DSM 20701^ T^*.* TR, trace (amounts < 1%); -, not detected. *Data were obtained from the original descriptions of species

### Genome properties and comparison

**Fig. 2:**
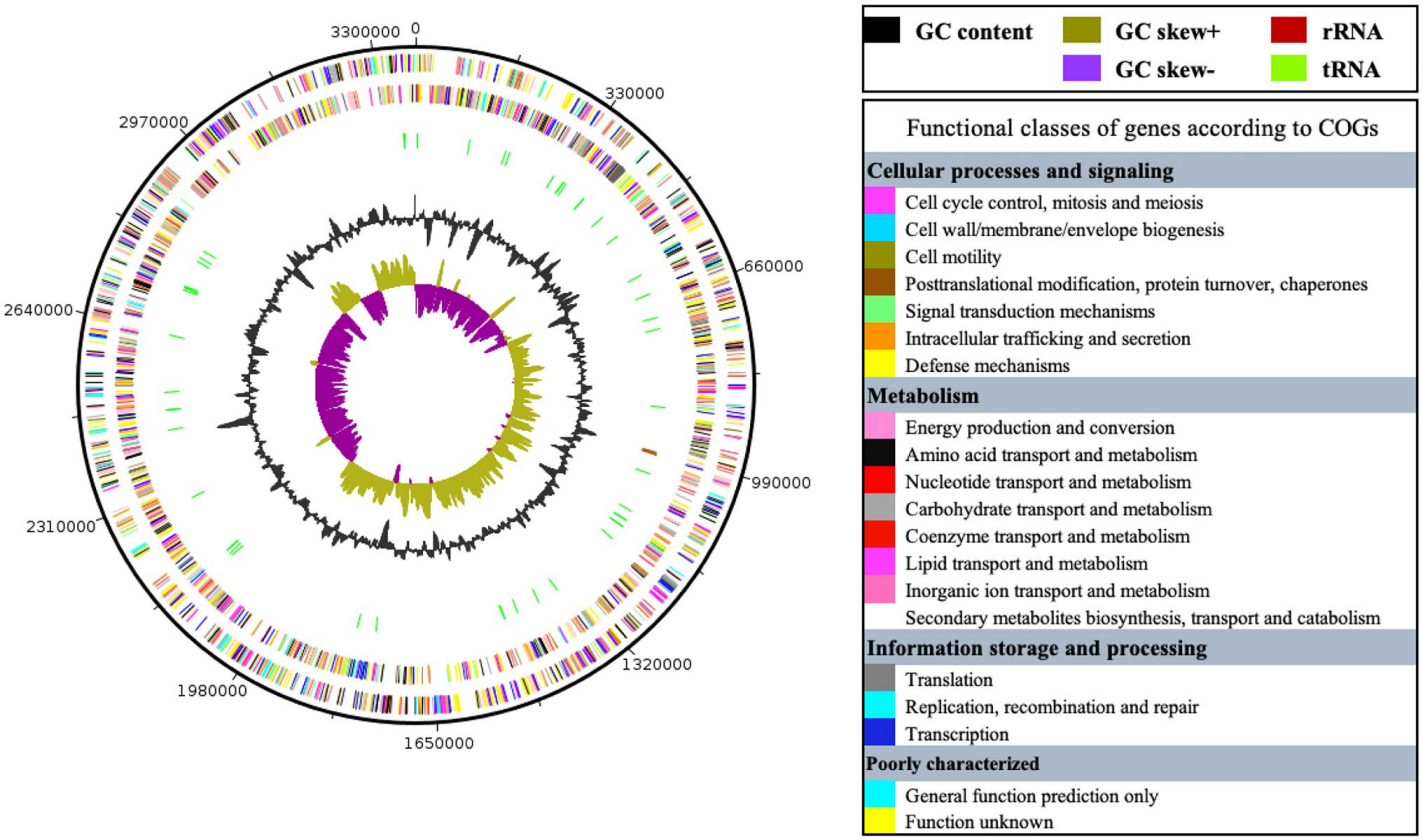
Graphical circular map of the chromosome of strain KD22.^T^. From the outside to the center: genes on the forward strand colored by Clusters of Orthologous Groups of proteins (COG) categories (only genes assigned to COG), genes on the reverse strand colored by COG categories (only gene assigned to COG), RNA genes (tRNAs green and rRNAs red), GC content (dark), and GC skew (negative values purple and positive values olive green)

**Fig. 3:**
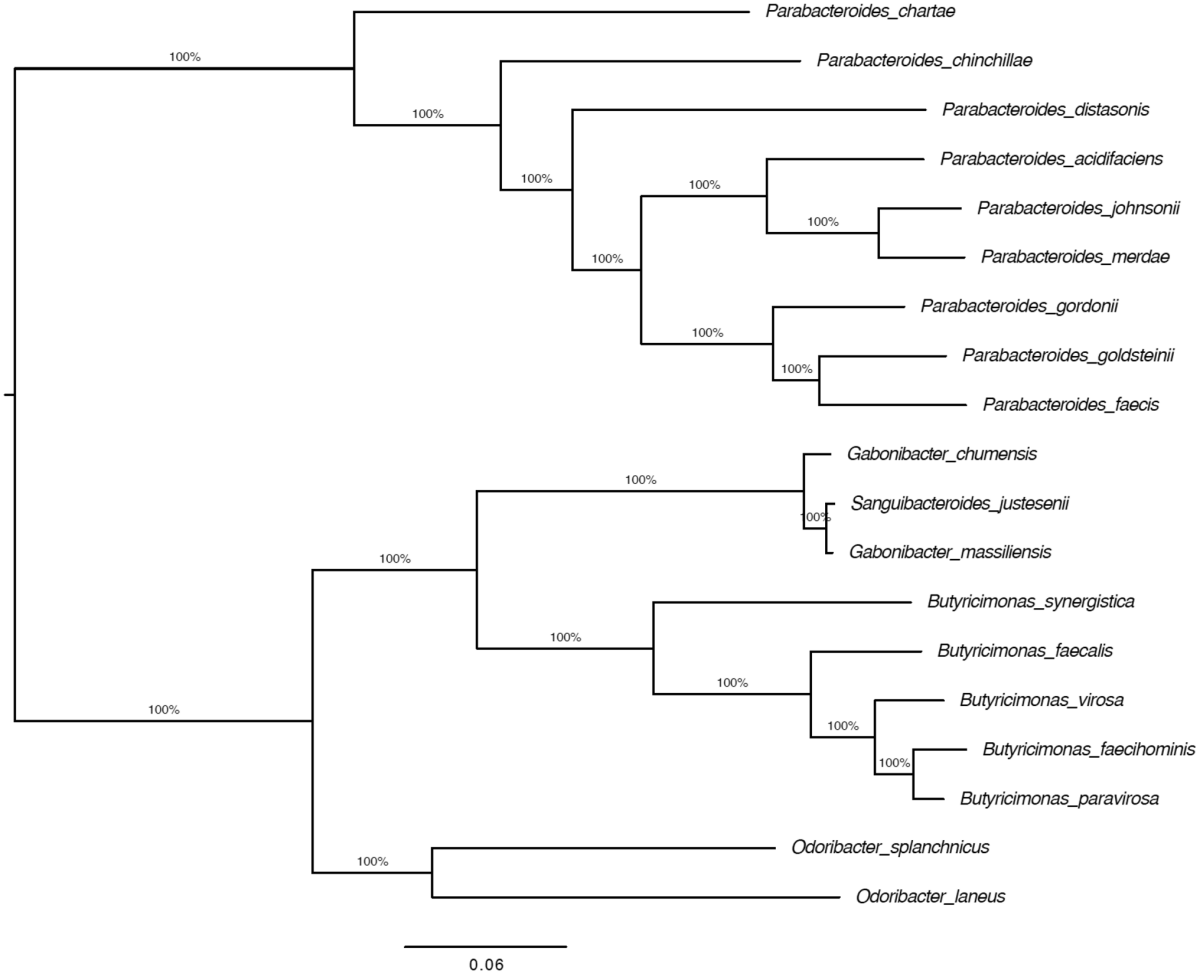
Core-genome phylogenetic tree between strain KD22^T^ and other related species. Phylogenetic tree built with a concatenated alignment of 108 single copy genes present in all the analyzed genomes, using the maximum-likelihood method with GTR model. Percentage bootstrap support values above 99% are shown for each node. The bar is nucleotide substitutions per site

After assembly, filtered reads of the draft genome of KD22^T^ resulted in 14 scaffolds (composed of 14 contigs) with a total sequence length of 3,368,578 bp (Table S1, Fig. 2). The repartition of genes into the 25 general COG categories is represented in Table S2. The genome DNA G + C content of strain KD22^T^ was 41.99 mol%. Of the 2,827 predicted genes, 2,746 were protein-coding genes and 59 were RNAs (one 5S rRNA, one 16S rRNA, one 23S rRNA, 53 tRNAs, and 3 ncRNAs genes). The genomic comparison of KD22^T^ with its neighbors is presented in Fig. 3, Table 3 and Table S1. Briefly, the genome size, gene content, and DNA G + C content of KD22^T^ (3.37 Mb, 2,827, and 41.99 mol%, respectively) are in the range reported for type strains of phylogenetically related species but very close to those of *G. massiliensis* (3.39 Mb, 2,880 and 42.10 mol% respectively; Table S1). Strain KD22^T^ and *G. massiliensis show high level of AAI values* (96.03%, Table S3). Nevertheless, the dDDH values between species ranged from 18.70% with ‘*S. justeseni’i and O. laneus* to 84.40% between *G. massiliensis* and ‘*S. justesenii’*. Strain KD22^T^ shared dDDH values from 18.50% with *Butyricimonas virosa* to 67.40% with *G. massiliensis* (Table 3). Furthermore, all these compared genomes had less than 97.50% ANI values (between *G. massiliensis* and ‘*S. justesenii’*). Strain KD22^T^ shared ANI values ranging from 65.63% with *Parabacteroides distasonis* to 95.43% with *G. massiliensis* (Table 3).

**Table 3:**
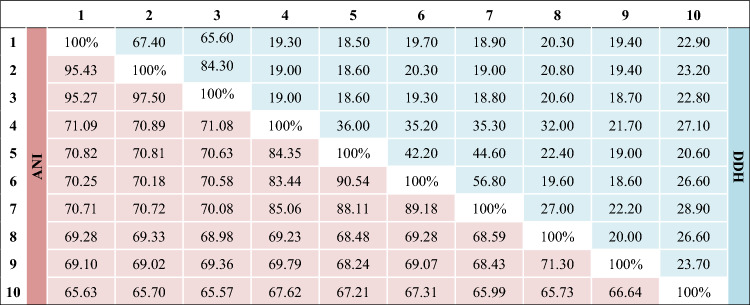
Genomic comparison between *Gabonibacter chumensis* KD22^T^ and closest related species using Pairwise comparisons, such as DDH (upper right side in blue) and ANI values (lower left side in read)

Strains: 1, KD22^T^; 2, *Gabonibacter massiliensis* GM7^T^; 3, *Sanguibacteroides justesenii* OUH 308042^ T^; 4, *Butyricimonas faecalis* H184^T^; 5, *Butyricimonas virosa* MT12^T^; 6, *Butyricimonas faecihominis* 180-3^ T^; 7, *Butyricimonas paravirosa* 214-4^ T^; 8, *Odoribacter splanchnicus* DSM 20712^ T^*;* 9, *Odoribacter laneus* YIT 12061^ T^; 10, *Parabacteroides distasonis* DSM 20701^ T^

^a^ INSDC: International Nucleotide Sequence Database Collaboration

## Conclusion

A polyphasic approach based on a combination of 16S rRNA gene sequence analysis, phenotypic features, chemotaxonomic properties, and genomic data demonstrated that strain KD22^T^ belongs to the genus *Gabonibacter*. The AAI, 96.03%, is high between *G. chumensis* and *G. massiliensis* slightly above the 96% limit for differentiating two species. Note that it is appropriate to use the average amino acid identity (AAI) for more distant populations, because the resolution is progressively lost at the nucleotide level (Rodriguez-R and Konstantinidis 2014). However, its differing phenotypic and biochemical characteristics sustained by 16S rRNA gene sequence similarity, dDDH, and ANI values of 97.58%, 67.40%, and 95.43%, respectively, distinguished it from its closest relative species as well as other validly published members of the genus *Gabonibacter.* The above findings, 16S rRNA gene similarity < 98.65% (Kim et al. 2014; Yarza et al. 2014), dDDH < 70% (Auch et al. 2010), and ANI < 96% (Meier-Kolthoff et al. 2013; Chun et al. 2018), which are used for species delineation indicate that strain KD22^T^ represents a novel species within genus *Gabonibacter*. The name *Gabonibacter chumensis* sp. nov. is proposed for this new isolate. In addition, dDDH and ANI values between *G. massiliensis* GM7^T^ and ‘*S. justesenii’* OUH 308042^ T^ (84.40% and 97.50%, respectively) higher than the limit set for species demarcation suggest that these two strains belong to the same species.

### Taxonomic and nomenclature proposal

#### Description of *Gabonibacter chumensis* sp. nov.

*Gabonibacter chumensis* (chum.en’sis N.L. masc. adj. chumensis, referring to the acronym CHUM, meaning “Centre Hospitalier de l’Université de Montréal”, the clinical lab where the type strain was first isolated).

Cells are strictly anaerobic, Gram-negative, not mobile, and non-spore-forming coccobacilli with up to 0.5 µm in diameter and 1.5 µm in length. They occur as single rods or in a short chain. After 48 h anaerobically at 37 ºC on sheep blood-enriched Columbia agar, bacterial colonies are circular, white to pale cream with 0.6–1.7 mm of diameter. Growth occurs between 28 and 37 ºC with an optimum at 37 ºC. NaCl concentration and pH range allowing growth are 0–5% and 6–7, respectively. Catalase, oxidase, and urease are not produced. Indole is detected, and nitrate is not reduced to nitrites. Aesculin and gelatin are not hydrolyzed. Acid is produced from D-glucose, D-mannitol, salicin, D-mannose, D-sorbitol, and D-trehalose, but not from lactose, D-saccharose, D-maltose, D-xylose, L-arabinose, glycerol, D-cellobiose, D-melezitose, D-raffinose, and L-rhamnose. Using API® strips (ZYM and Rapid 32A), the strain exhibits alkaline phosphatase, acid pyroglutamic arylamidase, alanine arylamidase, α-chymotrypsin, esterase, esterase lipase, glutamyl acid glutamic, leucyl glycine arylamidase, and naphthol-AS-BI-phosphohydrolase activities. On the other hand, galactosidase (α and β), glucosidase (α and β), β-glucuronidase, N-acetyl-β-glucosaminidase, α-arabinosidase, α-manisidase, and α-fucosidase are negative. The most abundant fatty acids are iso-C_15:0_ and C_16:0_ 3-OH.

The type strain, KD22^T^ (= CSUR Q8104^T^ = DSM 115208), was isolated from the feces of a patient suffering from lung cell small cancer. Its draft genome measures 3,368,578 bp and exhibits a DNA G + C of 41.99 mol%. The GenBank/EMBL accession numbers of 16S rRNA gene and genome sequences are OP221267.1 and JANSKB000000000.1, respectively.

### Supplementary Information

Below is the link to the electronic supplementary material.

Supplementary file 1—Figure S1. Main spectra library (MSP) dendrogram of MALDI-TOF mass spectral profiles from strain KD22 and its neighbors generated by the MALDI Biotyper 3.0 software. Distance is displayed in relative units.

Supplementary file 2—Figure S2. Tamura-Nei model maximum likelihood phylogenetic tree based on 16S rRNA gene sequences, showing the relationships between strain KD22T and closest related taxa. GenBank accession numbers are shown in parentheses. Numbers at nodes indicate bootstrap percentages (based on 100 replicates). Bar, 0.05 substitutions per nucleotide position.

Supplementary file 3—Figure S3. Transmission electron microscopy of *Gabonibacter* *chumensis* strain KD22T using a 375 Tecnai G20 transmission electron microscope.

Supplementary file 4—Table S1. Genome comparison of closely related species to strain KD22T.

Supplementary file 5—Table S2. Number of genes associated with the 25 general COG functional categories.

Supplementary file 6—Table S3. Average amino acid identity (AAI) between *Gabonibacter* *chumensis* KD22T and closest related species.

## Data Availability

The whole genome of *Gabonibacter chumensis* KD22^T^ has been deposited in NCBI GenBank database under the accession number JANSKB000000000.1. The Genbank accession number of the 16S rRNA gene sequence of strain KD22^T^ is OP221267.1.

## Abbreviations

ANI: Average nucleotide identity
CHUM: University of Montreal Healthcare Centre
CSUR: *Collection de souches de l’unité des Rickettsies*
dDDH: Digital DNA–DNA Hybridization
DSM: *Deutsche Sammlung von Mikroorganismen*
MALDI-TOF: Matrix-assisted laser desorption/ionization time-of-flight
MS: Mass spectrometry
NSCLC: Non-small cell lung cancer
