# Supplementary figures and images for: Characterization and description of Gabonibacter chumensis sp. nov., isolated from feces of a patient with non-small cell lung cancer treated with immunotherapy
Source: Arch Microbiol. 2023 Sep 24;205(10):338. doi: 10.1007/s00203-023-03671-0 (PMC10518271; doi:10.1007/s00203-023-03671-0)

MSP Dendrogram

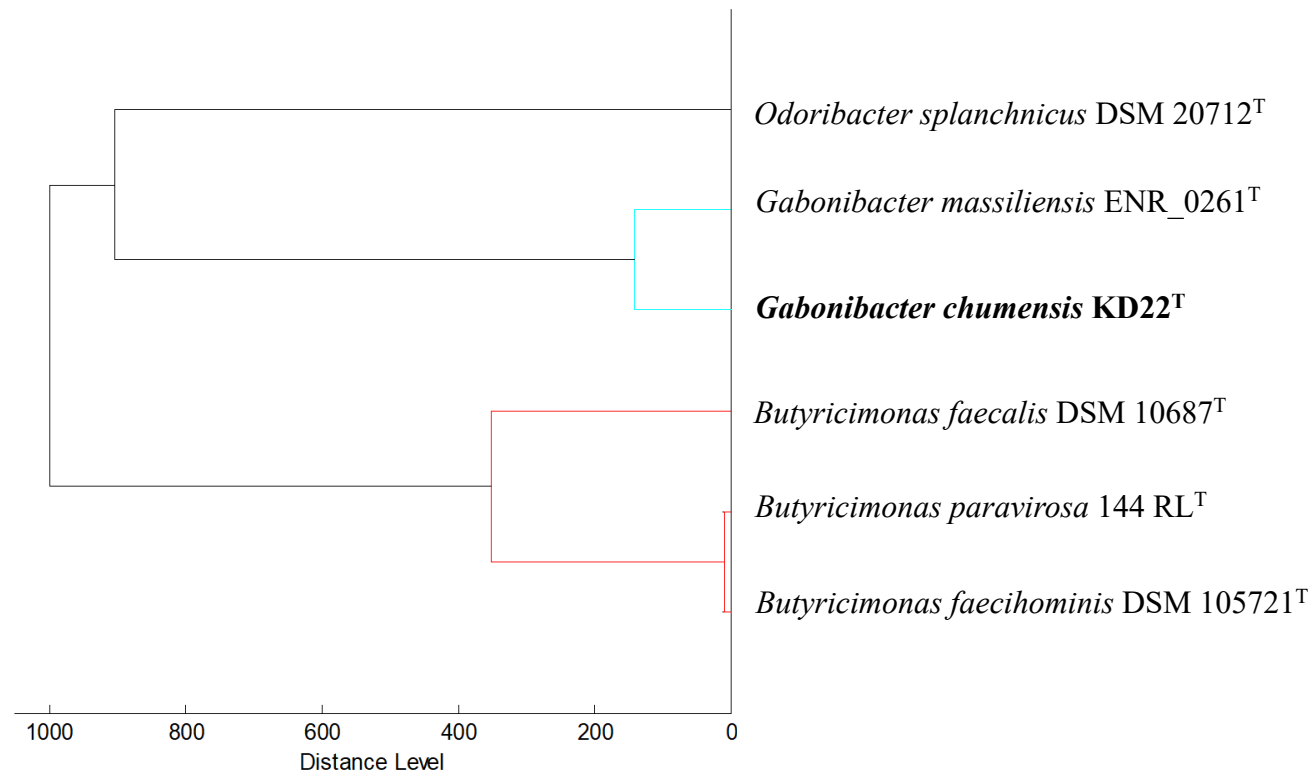

Supplement: Supplementary file 1 — Figure S1. Main spectra library (MSP) dendrogram of MALDI-TOF mass spectral profiles from strain KD22 and its neighbors generated by the MALDI Biotyper 3.0 software. Distance is displayed in relative units. [file 203_2023_3671_MOESM1_ESM.pdf]

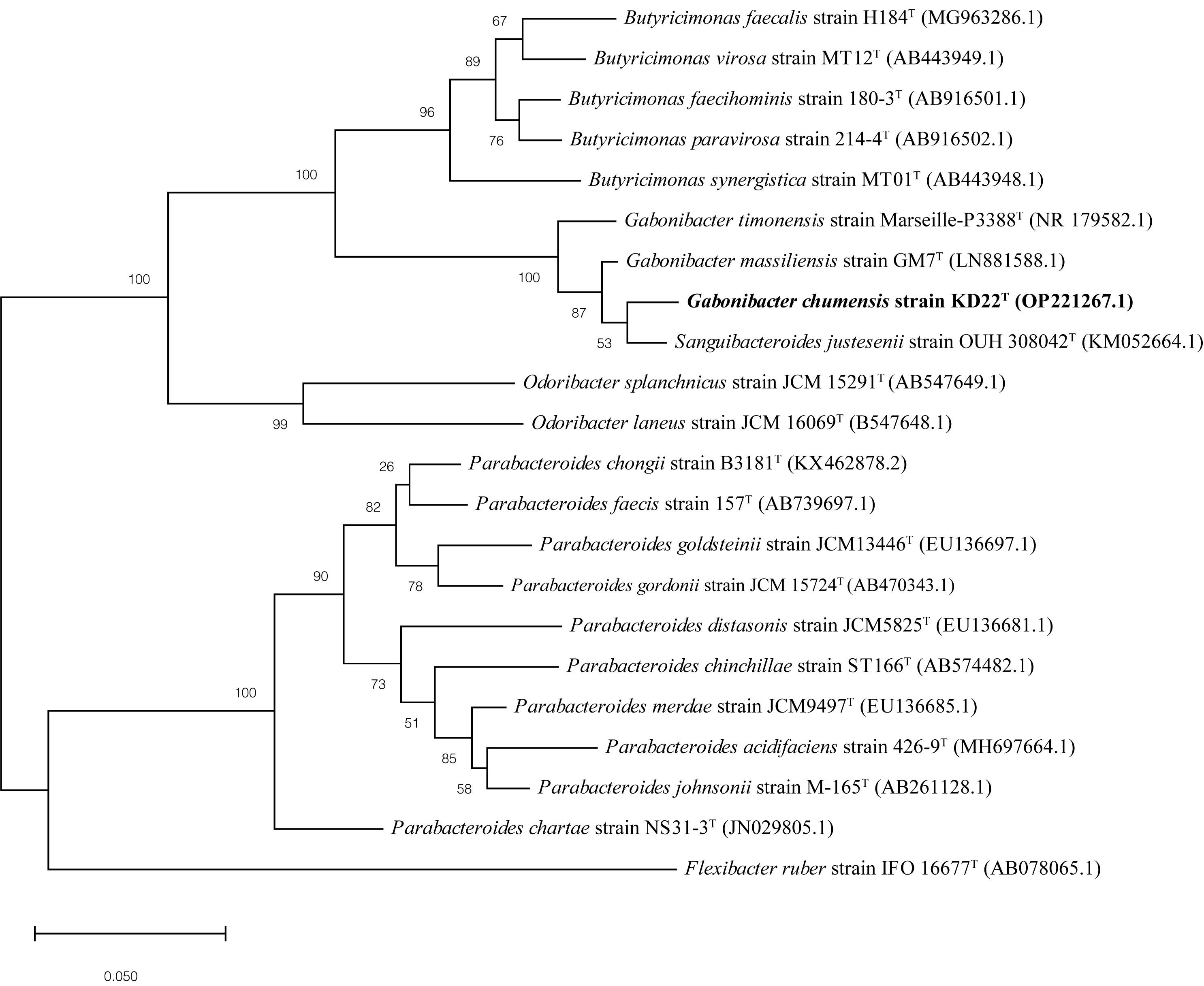

Supplement: Supplementary file 2 — Figure S2. Tamura-Nei model maximum likelihood phylogenetic tree based on 16S rRNA gene sequences, showing the relationships between strain KD22T and closest related taxa. GenBank accession numbers are shown in parentheses. Numbers at nodes indicate bootstrap percentages (based on 100 replicates). Bar, 0.05 substitutions per nucleotide position. [file 203_2023_3671_MOESM2_ESM.tif]

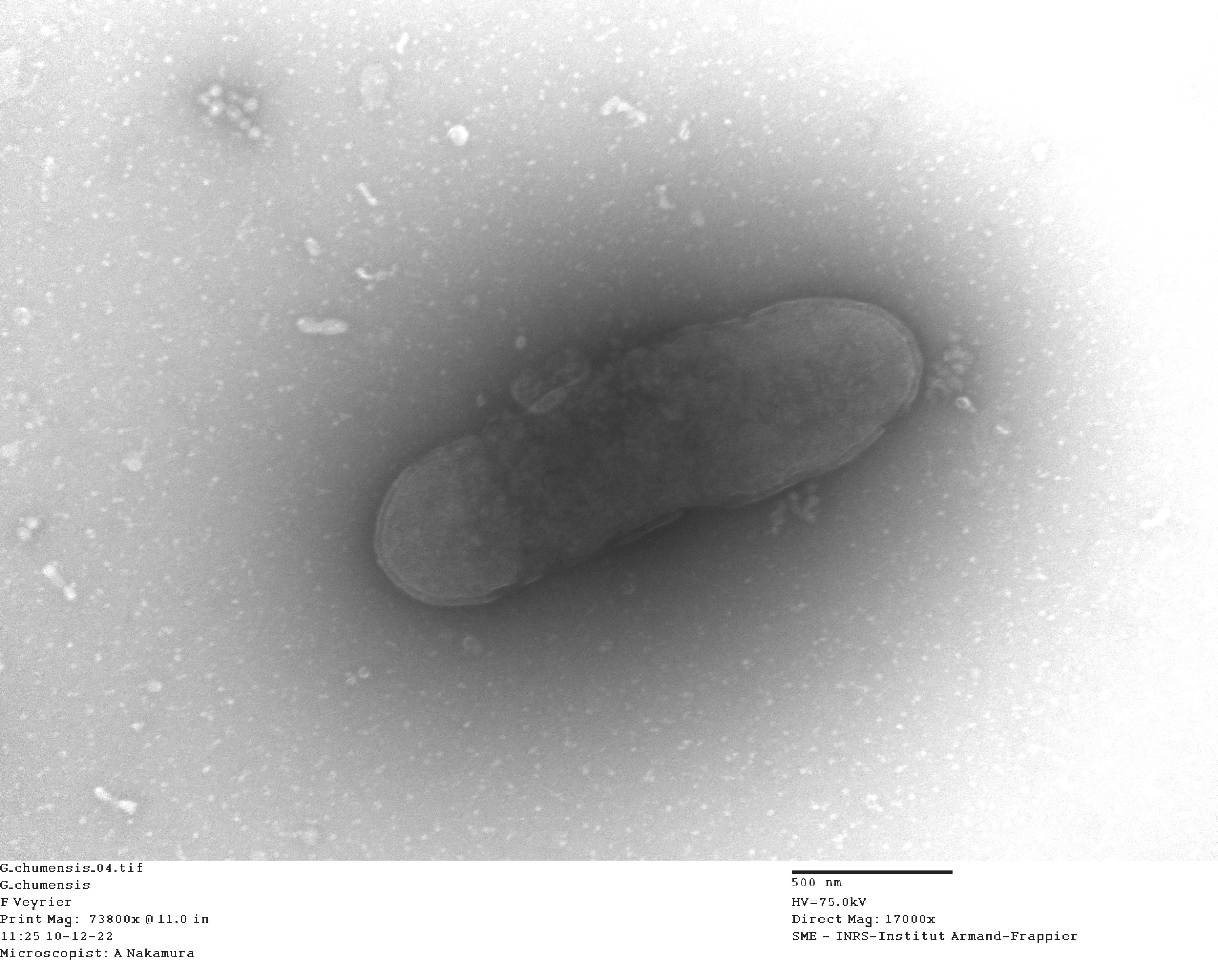

Supplement: Supplementary file 3 — Figure S3. Transmission electron microscopy of Gabonibacter chumensis strain KD22T using a 375 Tecnai G20 transmission electron microscope. [file 203_2023_3671_MOESM3_ESM.jpg]
