# Supplementary material for: Characterization and description of Gabonibacter chumensis sp. nov., isolated from feces of a patient with non-small cell lung cancer treated with immunotherapy
Source: Arch Microbiol. 2023 Sep 24;205(10):338. doi: 10.1007/s00203-023-03671-0 (PMC10518271; doi:10.1007/s00203-023-03671-0)
Supplement: Supplementary file 4 — Table S1. Genome comparison of closely related species to strain KD22T. [file 203_2023_3671_MOESM4_ESM.docx]

| **Species** | **Strain** | **^a^INSDC identifier** | **Size (Mb)** | **GC Percent** | **Gene Content** | **Protein** |
| --- | --- | --- | --- | --- | --- | --- |
| *Gabonibacter chumensis* | KD22 | JANSKB000000000.1 | 3.37 | 41.99 | 2,827 | 2,746 |
| *Gabonibacter massiliensis* | GM7 | FAVK00000000.1 | 3.39 | 42.10 | 2,880 | 2,818 |
| *Sanguibacteroides justesenii* | OUH 308042 | JPIU00000000.1 | 3.39 | 42.3 | 2,746 | 2,678 |
| *Butyricimonas faecalis* | H184 | CP032819.1 | 4.92 | 43.60 | 4,215 | 4,084 |
| *Butyricimonas virosa* | MT12 | JAEW00000000.1 | 4.72 | 42.30 | 3,925 | 3,813 |
| *Butyricimonas faecihominis* | 180-3 | BMOZ00000000.1 | 4.79 | 42.90 | 3,952 | 3,845 |
| *Butyricimonas paravirosa* | 214-4 | BMPA00000000.1 | 5.54 | 42.90 | 4,491 | 4,397 |
| *Odoribacter splanchnicus* | DSM 20712 | CP002544.1 | 4,39 | 43.40 | 3,710 | 3,545 |
| *Odoribacter laneus* | YIT 12061 | ADMC00000000.1 | 3.77 | 40.60 | 3,100 | 2,993 |
| *Parabacteroides distasonis* | DSM 20701 | CP050956.1 | 5.11 | 45.10 | 4,230 | 4,067 |

**Table S1.** Genome comparison of closely related species to strain KD22^T^

^a^ INSDC: International Nucleotide Sequence Database Collaboration
