# Supplementary material for: Characterization and description of Gabonibacter chumensis sp. nov., isolated from feces of a patient with non-small cell lung cancer treated with immunotherapy
Source: Arch Microbiol. 2023 Sep 24;205(10):338. doi: 10.1007/s00203-023-03671-0 (PMC10518271; doi:10.1007/s00203-023-03671-0)
Supplement: Supplementary file 5 — Table S2. Number of genes associated with the 25 general COG functional categories. [file 203_2023_3671_MOESM5_ESM.docx]

Table S2. Number of genes associated with the 25 general COG functional categories

| **Code** | **Value** | **% of total** | **Description** |
| --- | --- | --- | --- |
| [J] | 154 | 5,61 | Translation |
| [A] | 1 | 0,04 | RNA processing and modification |
| [K] | 148 | 5,39 | Transcription |
| [L] | 136 | 4,95 | Replication, recombination, and repair |
| [B] | 0 | 0,00 | Chromatin structure and dynamics |
| [D] | 32 | 1,17 | Cell cycle control, mitosis, and meiosis |
| [Y] | 0 | 0,00 | Nuclear structure |
| [V] | 77 | 2,81 | Defense mechanisms |
| [T] | 75 | 2,73 | Signal transduction mechanisms |
| [M] | 229 | 8,34 | Cell wall/membrane biogenesis |
| [N] | 27 | 0,98 | Cell motility |
| [Z] | 2 | 0,07 | Cytoskeleton |
| [W] | 0 | 0,00 | Extracellular structures |
| [U] | 37 | 1,35 | Intracellular trafficking and secretion |
| [O] | 84 | 3,06 | Post‐translational modification, protein turnover, chaperones |
| [X] | 0 | 0,00 | Mobilome: prophages, transposons |
| [C] | 171 | 6,23 | Energy production and conversion |
| [G] | 71 | 2,59 | Carbohydrate transport and metabolism |
| [E] | 197 | 7,18 | Amino acid transport and metabolism |
| [F] | 77 | 2,81 | Nucleotide transport and metabolism |
| [H] | 105 | 3,83 | Coenzyme transport and metabolism |
| [I] | 61 | 2,22 | Lipid transport and metabolism |
| [P] | 168 | 6,12 | Inorganic ion transport and metabolism |
| [Q] | 13 | 0,47 | Secondary metabolites biosynthesis, transport, and catabolism |
| [R] | 0 | 0,00 | General function prediction only |
| [S] | 566 | 20,62 | Function unknown |
| _ | 179 | 6,52 | Not in COGs |
