# Supplementary material for: Characterization and description of Gabonibacter chumensis sp. nov., isolated from feces of a patient with non-small cell lung cancer treated with immunotherapy
Source: Arch Microbiol. 2023 Sep 24;205(10):338. doi: 10.1007/s00203-023-03671-0 (PMC10518271; doi:10.1007/s00203-023-03671-0)
Supplement: Supplementary file 6 — Table S3. Average amino acid identity (AAI) between Gabonibacter chumensis KD22T and closest related species. [file 203_2023_3671_MOESM6_ESM.docx]

**Table S3.** Average amino acid identity (AAI) between *Gabonibacter chumensis* KD22^T^ and closest related species using Pairwise comparisons such as DDH (upper right side in blue) and ANI values (lower left side in read).

|  |  | **1** | | **2** | | **3** | | **4** | | **5** | | **6** | | **7** | | **8** | | **9** | | 10 |
| --- | --- | --- | --- | --- | --- | --- | --- | --- | --- | --- | --- | --- | --- | --- | --- | --- | --- | --- | --- | --- |
| **1** | 100% | |  | |  | |  | |  | |  | |  | |  | |  | |  | |
| **2** | 96,20 | | 100% | |  | |  | |  | |  | |  | |  | |  | |  | |
| **3** | 95.42 | | 97.93 | | 100% | |  | |  | |  | |  | |  | |  | |  | |
| **4** | 70.00 | | 69.87 | | 69.87 | | 100% | |  | |  | |  | |  | |  | |  | |
| **5** | 70.71 | | 70.69 | | 7085 | | 87.18 | | 100% | |  | |  | |  | |  | |  | |
| **6** | 71.01 | | 71.25 | | 71.38 | | 86.70 | | 91.01 | | 100% | |  | |  | |  | |  | |
| **7** | 70.66 | | 70.76 | | 7097 | | 8724 | | 90.78 | | 92.82 | | 100% | |  | |  | |  | |
| **8** | 63.40 | | 63.10 | | 63.64 | | 62.96 | | 62.16 | | 61.88 | | 62.42 | | 100% | |  | |  | |
| **9** | 63.49 | | 63.69 | | 64.11 | | 63.06 | | 62.60 | | 62.60 | | 63.45 | | 66.20 | | 100% | |  | |
| **10** | 49.87 | | 50.83 | | 50.35 | | 49.2 | | 49.77 | | 49.97 | | 49.98 | | 50.29 | | 51.50 | | 100% | |

Strains: 1, KD22^T^ ; 2, *Gabonibacter massiliensis* GM7^T^ ; 3, *Sanguibacteroides justesenii* OUH 308042^T^ ; 4, *Butyricimonas faecalis* H184^T^ ; 5, *Butyricimonas virosa* MT12^T^ ; 6, *Butyricimonas faecihominis* 180-3^T^ ; 7, *Butyricimonas paravirosa* 214-4^T^ ; 8, *Odoribacter splanchnicus* DSM 20712^T^ *;* 9, *Odoribacter laneus* YIT 12061^T^ ; 10, *Parabacteroides distasonis* DSM 20701^T^*.*
